# Exploring the Effect of Rotenone—A Known Inducer of Parkinson’s Disease—On Mitochondrial Dynamics in *Dictyostelium discoideum*

**DOI:** 10.3390/cells7110201

**Published:** 2018-11-08

**Authors:** Ethan Chernivec, Jacie Cooper, Kari Naylor

**Affiliations:** Department of Biology, University of Central Arkansas, Conway, AR 72035, USA; echernivec1@cub.uca.edu (E.C.); jacielcooper@gmail.com (J.C.)

**Keywords:** mitochondrial dynamics, fission, fusion, motility, Parkinson’s disease, ROS

## Abstract

Current treatments for Parkinson’s disease (PD) only alleviate symptoms doing little to inhibit the onset and progression of the disease, thus we must research the mechanism of Parkinson’s. Rotenone is a known inducer of parkinsonian conditions in rats; we use rotenone to induce parkinsonian cellular conditions in *Dictyostelium discoideum*. In our model we primarily focus on mitochondrial dynamics. We found that rotenone disrupts the actin and microtubule cytoskeleton but mitochondrial morphology remains intact. Rotenone stimulates mitochondrial velocity while inhibiting mitochondrial fusion, increases reactive oxygen species (ROS) but has no effect on ATP levels. Antioxidants have been shown to decrease some PD symptoms thus we added ascorbic acid to our rotenone treated cells. Ascorbic acid administration suggests that rotenone effects may be specific to the disruption of the cytoskeleton rather than the increase in ROS. Our results imply that *D. discoideum* may be a valid cellular PD model and that the rotenone induced velocity increase and loss of fusion could prevent mitochondria from effectively providing energy and other mitochondrial products in high demand areas. The combination of these defects in mitochondrial dynamics and increased ROS could result in degeneration of neurons in PD.

## 1. Introduction

According to the CDC, from 2000 to 2013 the prevalence of Parkinson’s disease (PD) and accompanying death rates steadily increased [1]. In 2006, it was estimated that there were at least 500,000 cases of PD in the United States with an economic cost exceeding $6 billion per year [2]. Our goal is to better understand the mechanism of PD pathogenesis by studying mitochondrial dynamics in the model organism, *Dictyostelium discoideum*.

Current treatments for neurodegenerative diseases, like PD may alleviate the symptoms of the disease but do little to halt the progression of it. This drives the need for research which investigates the mechanisms that induce the onset or drive the progression of all neurodegenerative disease. Recent studies have shown that mutations in proteins responsible for removal of dysfunctional mitochondria contribute to the progression of Parkinson’s disease, perhaps because damaged mitochondria build up in the cell, ultimately disrupting cell function [3,4]. These studies have also shown that the mutations in these same proteins affect mitochondrial dynamics [3,5,6,7,8].

Functional mitochondria must be capable of routine dynamics to maintain structure and localization [9,10,11]. These dynamics, chiefly fission, fusion, and motility, determine the morphology and distribution of the mitochondria within the cell [12,13,14]. Mitochondrial motility is especially critical in large, highly polarized cells, such as neurons, where energy may be required at locations far from the cell body [11]. Defects in fission and fusion have been shown to decrease motility, most likely because of the tangling of fission/fusion deficient mitochondria [11,14]. It is also possible that decreased motility inhibits fission and fusion because of the inability of the organelles to move towards or away from each other [11,14]. Ultimately fission and fusion are necessary to maintain the health of the mitochondria. Damaged organelles can be broken off by fission and subsequently removed from the cell by mitophagy—the clearance of old and defective mitochondria. Alternatively, damaged mitochondria can be repaired by fusion, essentially by combining a healthy mitochondrion with a damaged one so that repair can take place [11,15,16].

Dysfunctional mitochondrial dynamics have been linked to multiple neurodegenerative diseases such as Charcot-Marie Tooth disease, Amyotrophic Lateral Sclerosis (ALS), Alzheimer’s disease, and Parkinson’s disease [17]. Parkinson’s disease is characterized by the progressive loss of dopaminergic neurons in the substantia nigra. The decrease in these neurons (and therefore dopamine) results in alkinesia, rigidity, tremors, and postural instability [18,19,20,21]. Though the exact mechanism for the onset and progression of the disease is not known, many recent studies have established a connection between mitochondrial dysfunction and Parkinson’s [3,7,8,11,20,22,23,24,25,26]. Specifically, it has been shown that inhibition of complex I in mitochondria leads to the destruction of dopamine neurons [27,28]. Interference with mitophagy also appears to promote PD [4,25,29]. This is caused by mutations in genes such as Parkin, PINK-1 and LRRK2, which contribute to the buildup of dysfunctional or damaged mitochondria [3,30,31]. The backlog of damaged mitochondria can trigger apoptosis and thus cell death. Furthermore, these mutant proteins also influence mitochondrial dynamics [7,8,24,25]. For example, LRRK2 localizes with Dlp1, the major regulator of mitochondrial fission [5] and parkin induces degradation of Mfn1/2, the major regulators of mitochondrial fusion, resulting in fragmented mitochondria [6]. In summary, alteration of mitochondrial function, degradation, and possibly dynamics plays a role in the onset and progression of Parkinson’s disease.

Studies have also shown that microtubule function affects mitochondria. Microtubules consist mostly of tubulin and are used as a transport mechanism for mitochondria. When mitochondria cannot be transported, ATP is not delivered efficiently throughout the cell inducing some cells to begin apoptotic processes. We have shown previously that the loss of microtubules decreases mitochondrial fission, fusion and motility [14,32]. Numerous researchers have also shown that depolymerization of microtubules induces degeneration of neurons [32,33,34,35]. Our work suggests that the loss of mitochondrial dynamics occurs prior to neurodegeneration [32]. In the absence of microtubules, mitochondria cannot move throughout the cell to provide for proper energy localization nor can they undergo fission or fusion for repair, resulting in the build-up of damaged organelles that possibly leads to neurodegeneration.

In an effort to better understand the role of mitochondrial dynamics in Parkinson’s disease we are using the model system, *Dictyostelium discoideum* [36] and the parkinsonian inducer rotenone. Rotenone is known to induce parkinsonian conditions in rats by preferentially targeting dopaminergic neurons [37,38]. It is known to inhibit complex I of the mitochondrial electron transport chain—causing a buildup of reactive oxygen species (ROS)—depolymerize microtubules, and ultimately degrade dopaminergic neurons [37,39,40]. Similarly, at the cellular level PD is characterized by an increase in ROS, a loss of complex I function, and depolymerization of microtubules [20].

*D. discoideum* is a well-established mitochondrial disease model [41] and a mitochondrial dynamics model [42]. In *D. discoideum*, mitochondria are spherical to tubule shaped organelles that undergo fission and fusion approximately 1 event every min [42]. These balanced events along with motility are dependent upon the cytoskeleton [14]. We take advantage of this model system to further explore mitochondrial dynamics under PD cellular conditions. There appears to be a link between mitochondrial dynamics and the onset/progression of neurodegenerative diseases such as Parkinson’s. If a link is established, new methods for identification and treatments of the disease are possible. In this study, we demonstrate that rotenone indeed affects mitochondrial dynamics, specifically by decreasing fusion and increasing mitochondrial velocity. These effects are likely not dependent on the increase in reactive oxygen species as a result of the rotenone treatment. This work further demonstrates the possible link between neurodegeneration and mitochondrial dynamics.

## 2. Materials and Methods

### 2.1. Strain Growth

The AX4 (wild-type) strain of *Dictyostelium discoideum* was obtained from Bill Loomis via Dicty Stock Center (www.dictybase.org). AX4 cells were cultured in liquid HL5 media containing streptomycin (300 µg/mL) and ampicillin (150 µg/mL) at 22 °C shaking at 125 rpm.

### 2.2. Experiment Preparation

Cells were diluted to 3 × 10^4^ cells/mL with HL5 media and grown until log phase (1–2 × 10^6^ cells/mL). The log phase cells were washed by centrifuging at 500× *g* for 4 min and resuspended in room temperature Lo-Flo (Formedium, Hunstanton, Norfolk, UK). MitoTracker CMXRos (Invitrogen, Thermo Fisher Scientific, Grand Island, NY, USA) (0.1 µM) was used to stain cells for 4 h at room temperature with shaking. The cells were washed two times using Lo-Flo (Formedium) in order to remove excess MitoTracker.

### 2.3. Rotenone Exposure

Freshly prepared rotenone (Sigma-Aldrich, St. Louis, MO, USA) was added during the last 2 h of the MitoTracker incubation period to a final concentration of 150 µM, rotenone’s LD_50_ in *D. discoideum*; an equal volume of DMSO was used as a vehicle control.

### 2.4. Ascorbic Acid Treatment

Cells to be treated with ascorbic acid were treated as above, then 2 mg/mL or 10 mg/mL ascorbic acid (or equivalent volume of dH_2_O) was added after rotenone exposure (prior to washing) for 3 h. Cells were washed two times with Lo-Flo as above.

### 2.5. Imaging

Cells were placed in Lab Tek II chambered cover glass slides and imaged using laser scanning LSM 510 Pascal confocal microscope (Zeiss, Stockholm, Sweden) with a pinhole setting of 144 µm (1.36 airy units), resulting in an optical slice of 1.1 µm. A single plane was imaged every 677.38 ms for 100 time points.

### 2.6. Immunofluorescence of Mitochondria and Cytoskeleton

15 mL of log phase AX4 were stained with MitoTracker CMXRos and treated with 150 μM rotenone or 150 μM rotenone and 10 mg/mL ascorbic acid as described above. After washing cells twice, with Lo-Flo, to remove excess MitoTracker, cells were resuspended to 4 × 10^6^ cells/mL and fresh rotenone, ascorbic acid, or vehicle was added. 0.5 mL of treated cells were added directly to a coverslip in a 6-well plate and incubated for 30 min at room temperature so the cells could attach. Cells were washed two times with 2 mL of 10 mM MES-NaOH solution then fixed with 1 mL of 3% paraformaldehyde pH 6.0 for 30 min. The cells were quenched with 1 mL of 100 mM glycine in 1× PBS for 5 min then permeabilized with 2 mL of 0.02% Triton X-100 for 5 min. After three quick 1× PBS washes, cells were incubated in 1 mL of 0.045% fish gelatin and 0.5% BSA in 1XPBS (PBG) for 1 h at room temperature. Mouse anti-tubulin (12G10, Developmental Studies Hybridoma Bank, Iowa City, IA, USA) diluted 1:150 in PBG solution was added to the coverslips and incubated at 4 °C 12–18 h. Three 5 min washes with 1× PBS were performed then the secondary antibody was added, AlexaFluor 488 goat alpha mouse IgG, (Life Tech, A11001, Thermo Fisher Scientific Grand Island, NY, USA) 1:250 in PBG solution for 1 h at 4 °C. The coverslips were kept in the dark from this point forward. Three final 5 min washes in 1× PBS were performed then coverslips were mounted to glass slides with SlowFade Gold (Invitrogen) and stored in the dark at 4 °C. To visualize actin, Alex-Fluor 488 Phalloidin (Invitrogen) diluted 1:100 in PBG was added to the coverslips for 1 h at room temperature, instead of the primary and secondary antibody steps. Coverslips were washed with 1× PBS and mounted as described above. Mitochondria and cytoskeletons were differentiated into categories based on morphology for greater than 40 cells per treatment. Cytoskeletons were differentiated into Full, Partial, or None. Full refers to a complete cytoskeletal network, Partial means a less complex structure; for microtubules this means shorter filaments, sometimes just the astral body itself. For actin this means patchy structures at the cell periphery. None refers to the cases where no fluorescence was visible. Mitochondria were classified as Dispersed- evenly spread throughout the cell, Clustered- distinct clusters of mitochondria—though there may be individuals, and Tight-, which means the majority of the organelles are found in tight clusters of mitochondria. In these cells this phenotype is not as clumped as a *cluA*-strain [43]. A Chi square analysis via Prism version 6.07 for Windows, (GraphPad, La Jolla, CA, USA, www.graphpad.com) was performed.

### 2.7. Quantification of Mitochondrial Velocity

ImageJ (freeware, 1.8.0_112, NIH, Bethseda, MD, USA) was used to create kymographs from the confocal microscope time-lapse images. Three kymographs were made for each cell, one for the left, middle, and right. These kymographs depict mitochondrial movement within the selected region over time. The rate pixel/time point, was converted to µmeters/s in order to be able to compare velocities. The velocities for the left, middle, and right of each cell were averaged, then the cell mean was calculated. The mean of 50 cells was analyzed using unpaired two tail *t*-test or Mann Whitney if data was non-parametric, via Prism version 6.07 for Windows, Graphpad software, La Jolla, CA, USA, www.graphpad.com. A *p*-value less than 0.05 was considered significant.

### 2.8. Quantification of Percent Motility

The same kymographs created above were analyzed for mitochondrial motility. Within each kymograph every line indicates an organelle, a vertical line indicates no movement by that specific organelle while zig-zags represent motility. The total lines were counted and the number of moving organelles was divided by the total number of organelles in order to create a percentage. Mann Whitney was used to analyze at least 50 cells for each treatment via Prism version 6.07 for Windows (Graphpad software, La Jolla, CA, USA, www.graphpad.com). A *p*-value less than 0.05 was considered significant.

### 2.9. Quantification of Fission and Fusion

Mitochondria must be present and visible in order to quantify fission and fusion. Image analysis was performed with LSM Imager Examiner software (3.2, Zeiss, Stockholm, Sweden). Fission was determined by a single organelle splitting into two organelles. Fusion was quantified by two mitochondria nearing each other for multiple frames and then fusing into one organelle [42]. The rates of fission and fusion were determined by calculating the average number of events/min/cell for a minimum of 30 cells in each treatment. Rates were compared using Kruskal-Wallis with Steel Dwass post-hoc analysis via JMP (12, SAS Institute, Cary, NC, USA). A *p*-value of less than 0.05 was considered statistically significant.

### 2.10. ATP Assay

ATP concentrations were determined using log phase cells treated with 150 µM rotenone, DMSO, or 150 µM rotenone plus 2 mg/mL ascorbic acid. 5 × 10^6^ cells in 1 mL of Lo-Flo were lysed by adding 150 µL of 50 mM Tris pH 8.5, 150 mM NaCl, 1% Triton, and approximately 100 µL glass beads. Cells were inverted three times then pelleted at 400× *g* for 10 min. 90 µL of supernatant with 10 µL of reaction mixture was used. Using Molecular Probes ATP Determination Kit (Thermo Fisher Scientific Grand Island, NY, USA), 5 independent experiments were conducted. Luminescence was read via BioTek’s (Winooski, VT, USA) Synergy H1 at 560 nm. Background luminescence was subtracted from all readings and a standard curve was created. ATP readings were done in triplicate then averaged. Before calculating sample ATP concentrations, cell lysate samples were normalized to a lysis buffer control. Prism version 6.07 for Windows, Graphpad software (La Jolla, CA, USA, www.graphpad.com) was used to carry out a paired *t*-test. A *p*-value less than 0.05 was considered significant.

### 2.11. ROS Assay

Log phase cells were pelleted and resuspended in SS6.4 Buffer Solution to 6 × 10^6^ cells/mL. Cells were treated with rotenone (0–500 µM), vehicle control of DMSO, or 10 mg/mL ascorbic acid plus rotenone for 2 h at 22 °C with shaking. 50 µL of cells were added into appropriate wells of a black 96-well plate in triplicate. 50 µL of 60 µM DHE was added to each well, fluorescence was measured using BioTek’s Synergy H1. Just before reading, 50 µL of untreated cells were mixed with 0.6 mM cumene hydroperoxide, (Sigma) as a positive control. Cells were excited at 522 nm and emission was measured at 605 nm for 10 min with a reading taking place every 30 s. The readings over the 10 min were averaged, then a mean for the triplicate readings was calculated. This mean was normalized to untreated (SS6.4 only) cells. This was repeated 4 times for ascorbic acid treatments and 6 times for rotenone treatments. Using Prism version 6.07 for Windows (Graphpad software, La Jolla, CA, USA, www.graphpad.com), a One-Way ANOVA with Tukey post hoc was used to determine the effect of rotenone on ROS. To determine if ascorbic acid decreased ROS after rotenone treatment a Paired two-tailed *t*-test was used. A *p*-value of 0.05 was considered significant.

## 3. Results

### 3.1. Rotenone Disrupts the Actin and Microtubule Cytoskeleton But Not Mitochondrial Morphology

**Figure 1:**
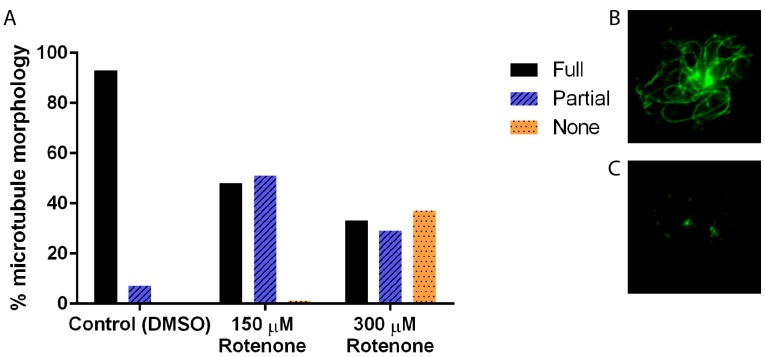
Microtubule morphology of cells treated with DMSO, 150 μM, or 300 μM of rotenone. (**A**) As the concentration of rotenone is increased, the percent of complete microtubule structures decreased (*p* < 0.001). Approximately 37% of cells treated with 300 μM rotenone had depolymerized microtubules (*n* ≥ 40). (**B**) Example of full microtubule structure, and (**C**) partial microtubule structure.

**Figure 2:**
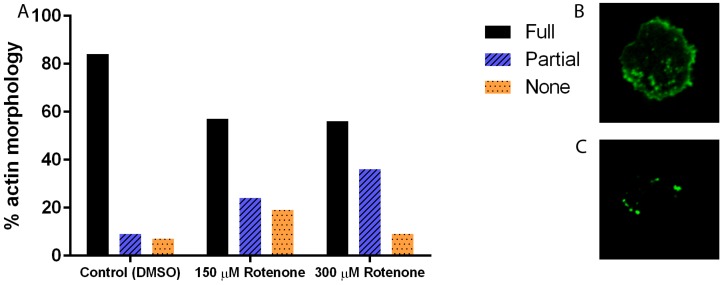
Percent actin morphology of cells treated with DMSO, 150 µM, or 300 µM of rotenone. (**A**) Increasing the concentration of rotenone decreased the complexity of the network and length of individual fibers (*p* = 0.0013). 84% of DMSO treated cells had full actin networks while 56% of 300 µM rotenone treated cells retained full actin networks (*n* ≥ 40). (**B**) Example of full actin cytoskeleton, and (**C**) partial actin cytoskeleton.

In many systems, including *D. discoideum*, the cytoskeleton is intricately linked to the processes of mitochondrial motility, fission, and fusion; thus to understand the effect of rotenone on these dynamics we must understand its effect on the cytoskeleton. Immunofluorescence analysis of the microtubule cytoskeleton demonstrated that increasing concentrations of rotenone, as expected, alters the microtubules, driving them toward less assembled shorter structures (*p* < 0.0001), with 93% of DMSO treated cells showing complete microtubule structures while only 33% of 300 µM rotenone treated cells showing complete structures (Figure 1). Unexpectedly, analysis of the actin filaments showed similar results: a decreased complexity of the network and length of individual fibers (*p* = 0.0013). 84% of DMSO treated cells had complex full actin networks while only 56% of 300 µM rotenone treated cells retained this structure (Figure 2).

To determine the effect of rotenone on mitochondrial morphology, the organelles were analyzed for change in shape, size, and clustering. There was no change in shape, size, or clustering, testing up to 300 µM of rotenone (data not shown).

### 3.2. Rotenone Stimulates Mitochondrial Velocity and Inhibits Mitochondrial Fusion

**Figure 3:**
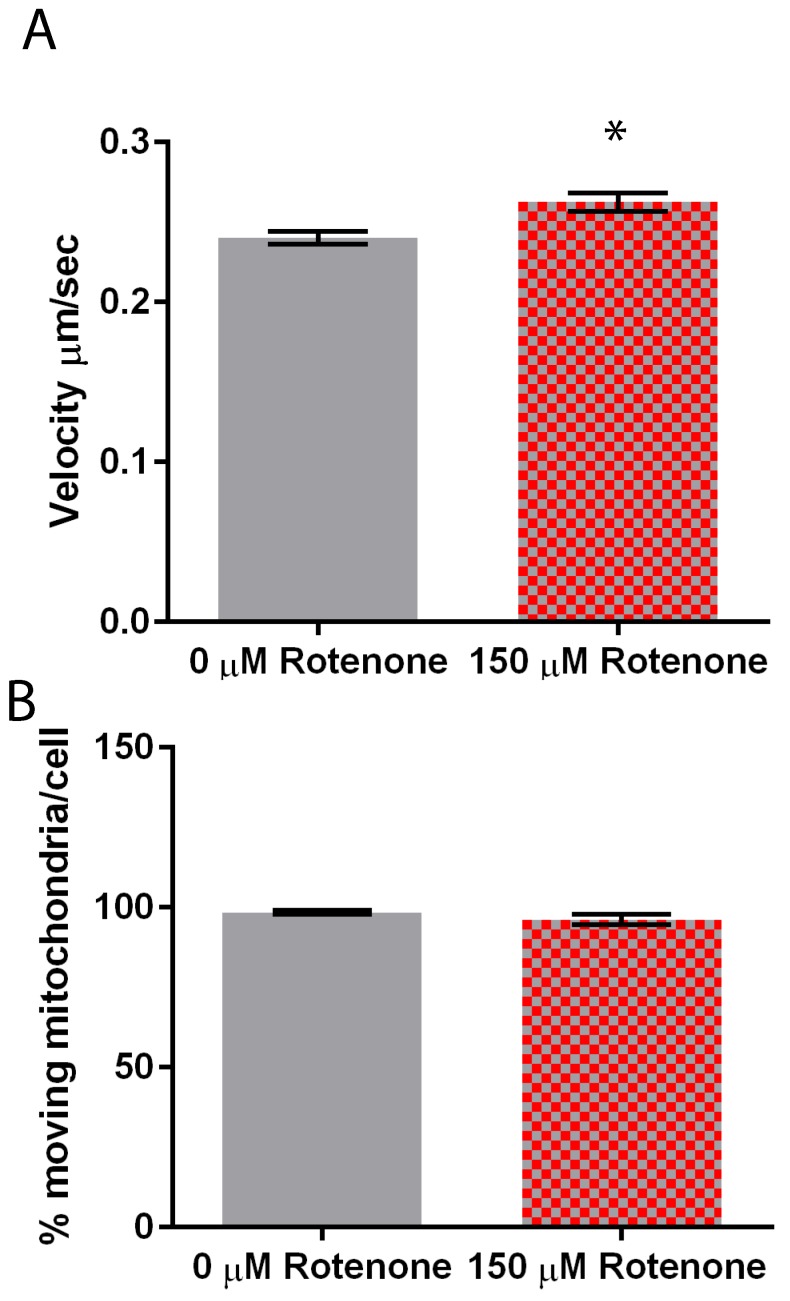
Mitochondrial motility in DMSO and rotenone treated cells. (**A**) Rotenone significantly increased velocity by 9.3% when compared to DMSO treated cells (*p* = 0.0016), (**B**) while the percent of moving mitochondria was unaffected. Sample size is *n* = 50 with standard error being represented by error bars on each column. * indicates significant difference between treatments.

To determine the effect of rotenone on mitochondrial velocity we treated cells with 150 µM rotenone or DMSO, the vehicle control. The velocity of mitochondria in rotenone treated cells was 0.2626 ± 0.006 µm/s, with the mitochondrial velocity in the control treatments being 0.2403 ± 0.004 µm/s (Figure 3A). Rotenone significantly increased mitochondrial velocity by 9.3% (*p* = 0.0016). Though the speed of the mitochondria was impacted by rotenone treatment the percent of mitochondria moving per cell remained unaffected. In control treatments mitochondria percent moving was 98.5% and in rotenone treated cells 96.2% of the organelles were moving (Figure 3B).

**Figure 4:**
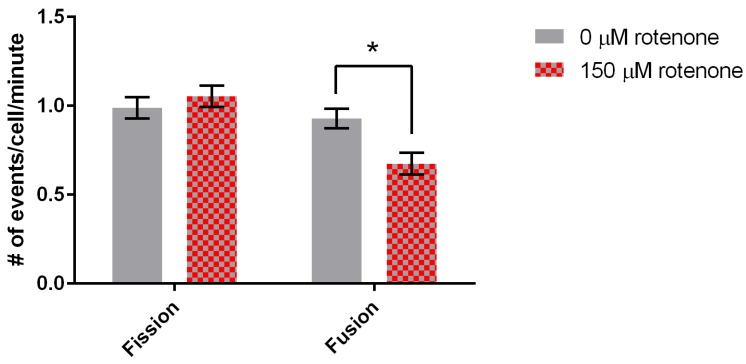
Fission and fusion rates for DMSO and rotenone treated cells. Fission was not significantly affected while fusion decreased 33% when exposed to rotenone (*p* = 0.0054), standard error is indicated by error bars (*n* = 88). * indicates significant difference between treatments.

Finally, we determined the effect of rotenone on mitochondrial fission and fusion. Mitochondria treated with 150 µM rotenone underwent fission at a rate of 1.05 ± 0.06 events/min, while when treated with DMSO fission was 0.99 ± 0.06 events/min, demonstrating no significant difference (Figure 4). Mitochondrial fusion in cells treated with DMSO was 0.93 ± 0.06 events/min. 150 µM rotenone treatment decreased fusion by 33% to 0.67 ± 0.06 events/min (*p* = 0.0054) (Figure 4). In summary, rotenone in *D. discoideum* increased mitochondrial velocity and decreased mitochondrial fusion.

### 3.3. Rotenone Increases ROS But Has Little Effect on ATP

Rotenone is known to decrease ATP and increase ROS [37,44]. To determine if rotenone behaves similarly in *D. discoideum* cells, ATP and ROS was quantified in cells exposed to rotenone.

**Figure 5:**
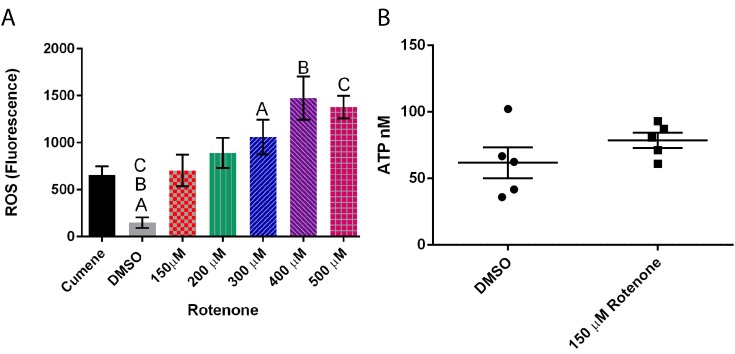
Effect of rotenone on reactive oxygen species (ROS) and ATP levels in *Dictyostelium discoideum*. (**A**) Increasing rotenone increases ROS (*n* = 6) (*p* = 0.0003), Columns with same letter are significantly different from each other, standard error is represented by the error bars on each column. (**B**) Addition of 150 μM rotenone had no significant effect on ATP levels (*n* = 5). The scatter columns represent all data points, average, and standard error.

Exposing cells to a range of rotenone (0 to 500 µM) demonstrated that increasing rotenone concentrations to 400 μM increased the presence of ROS by almost 900% (*p* = 0.0003) (Figure 5A). Interestingly, rotenone had no effect on ATP levels (Figure 5B), thus the effects of rotenone on mitochondrial dynamics may be caused by the increase in ROS but not due to ATP changes.

### 3.4. Ascorbic Acid Returns the Cytoskeleton to Normal

Alteration of mitochondrial dynamics is implicated in a variety of neurological diseases, but the role of these changes is unclear. In this study we used a known inducer of PD in a model system to explore if alterations in mitochondrial dynamics is a real factor. As rotenone does alter dynamics in our model system, this suggests that the dynamics are more important than we previously thought. So, how does rotenone cause these changes? Ascorbic acid is a well-established antioxidant [45] thus we added ascorbic acid to our rotenone treated cells to determine if we could return the cells back to a wild-type phenotype.

**Figure 6:**
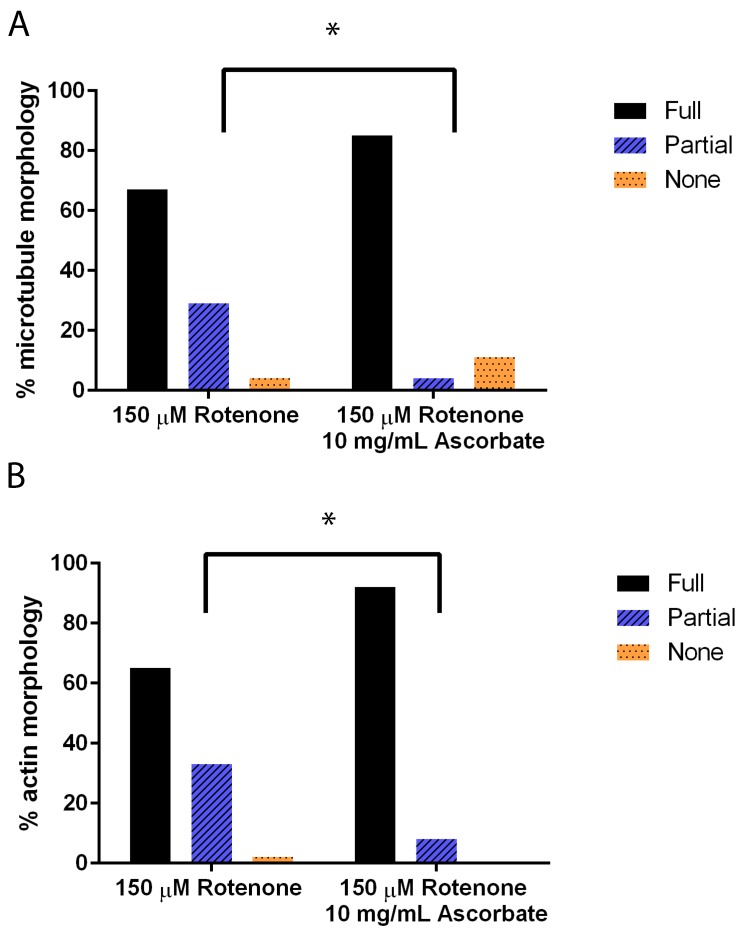
Cytoskeleton morphology in cells treated with rotenone and 0 mg/mL or 10 mg/mL of ascorbic acid. (**A**) Microtubule structures were significantly more assembled in cells when treated with ascorbic acid than rotenone-treated alone (*p* = 0.0017). (**B**) Actin networks were significantly more assembled in cells when treated with ascorbic acid than rotenone-treated alone (*n* ≥ 40) (*p* = 0.0032). * indicates significant difference between treatments.

Again using immunofluorescence to analyze the cytoskeleton, we added 10 mg/mL of ascorbic acid to cells treated previously with rotenone. This induced the microtubules (*p* = 0.0017) and actin (*p* = 0.0032) filaments to be significantly more assembled than rotenone treated alone (Figure 6).

**Figure 7:**
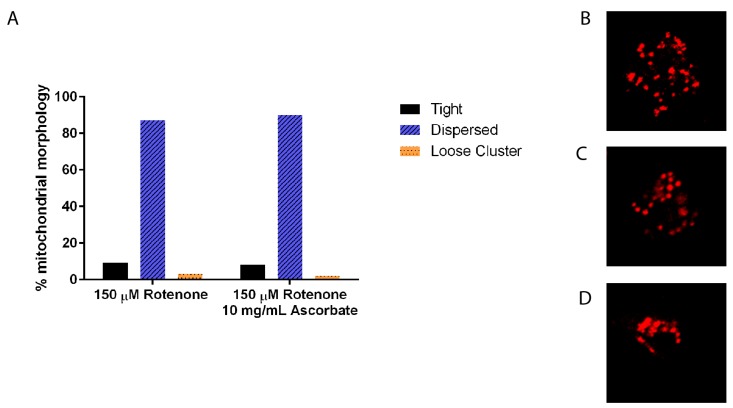
Mitochondrial morphology in cells treated with rotenone and 0 mg/mL or 10 mg/mL of ascorbate acid. (**A**) There was no significant morphology difference in mitochondria treated with ascorbate acid than rotenone treatment alone (*n* ≥ 80). Standard error is indicated by error bars. (**B**) Example of fully dispersed mitochondria, (**C**) loose cluster, and (**D**) tight clusters of mitochondria.

To ensure that 10 mg/mL ascorbic acid does not have an independent effect on mitochondrial morphology, again size, shape, and clustering was analyzed. There was no difference between 150 µM rotenone and 150 µM rotenone + 10 mg/mL ascorbic acid in size and shape (data not shown) nor in clustering with 87% and 90% of cells having fully dispersed mitochondria, respectively (Figure 7).

### 3.5. Ascorbic Acid Continues to Increase Velocity But Returns Fusion to Normal

As described above, 150 µM rotenone unbalanced the processes of fission and fusion, effectively decreasing the fusion events but increasing the rate of mitochondrial movement. To determine if this is dependent on the increased ROS we added 10 mg/mL ascorbic acid to the rotenone treated cells and measured mitochondrial dynamics.

**Figure 8:**
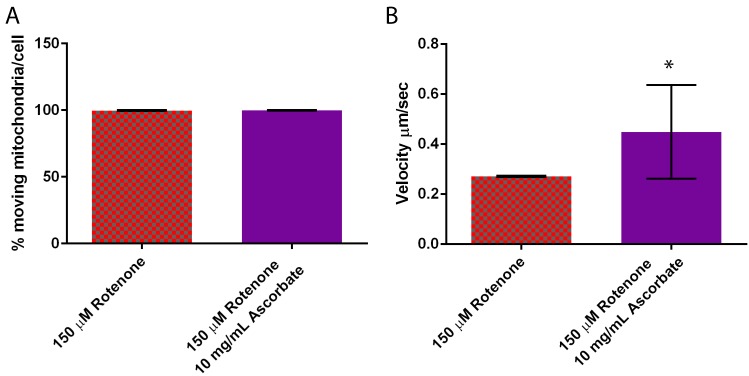
Mitochondrial motility in cells treated with rotenone and the antioxidant ascorbic acid. (**A**) Percent moving mitochondria remained unaffected, (**B**) while ascorbic acid significantly increased velocity by 55% when compared to rotenone treated cells (*p* = 0.0131) (*n* = 50). Standard error is represented by error bars. * indicates significant difference between treatments.

The percent of mitochondria moving per cell remained unaffected by the 10 mg/mL ascorbic acid treatment averaging 98–100% moving (Figure 8A), though the ascorbic acid treatment increased velocity 55% beyond the increase already induced by rotenone from 0.27 ± 0.003 µm/s to 0.45 ± 0.19 µm/s (*p* = 0.0131) (Figure 8B). Interestingly a 2 mg/mL ascorbic acid treatment had no effect on velocity when compared to 150 µM rotenone (data not shown).

**Figure 9:**
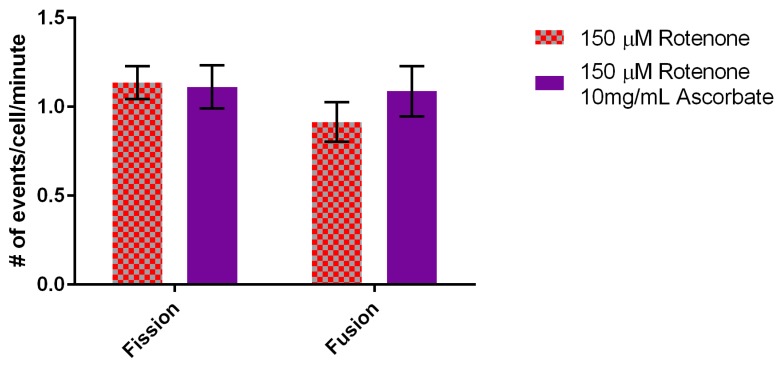
Effect of rotenone and 10 mg/mL ascorbic acid on mitochondrial fission and fusion rates. 10 mg/mL ascorbic acid increased fusion rates back to normal levels (statistically no significant difference) when compared to rotenone treated cells (*n* = 30). Error bars represent standard error.

In terms of fusion, 2 mg/mL of ascorbic acid did not overcome the fusion defect induced by rotenone (data not shown) but addition of 10 mg/mL ascorbic acid did return fusion rates back to normal levels of 1.09 ± 0.14 events/min (Figure 9). In conclusion, the addition of 10 mg/mL ascorbic acid to rotenone treated cells increased mitochondrial velocity beyond the response to rotenone yet returned fusion levels back to normal.

### 3.6. Ascorbic Acid Does Not Alter ATP Levels but It Does Slightly Decrease ROS Levels

**Figure 10:**
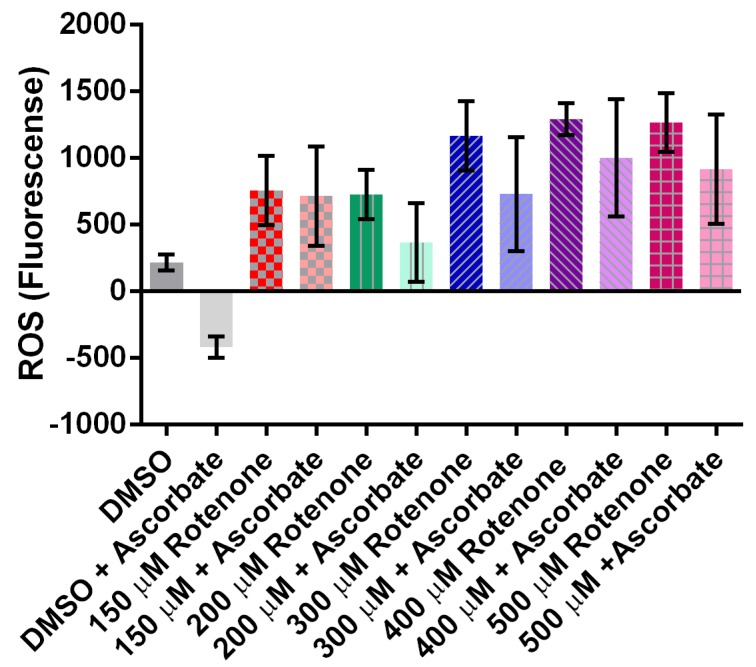
The effect of ascorbic acid on rotenone induced ROS. 10 mg/mL of ascorbic acid lowered ROS but not significantly (*n* = 4). Error bars represent standard error.

We measured ATP levels in cells exposed to rotenone and 2 mg/mL ascorbic acid. There was no significant difference between ATP in rotenone treated cells (172.4 ± 98.48 nM) and the ascorbic acid treated cells (156.9 ± 50.16 nM). Interestingly, 10 mg/mL of ascorbic acid was not enough to return ROS levels to the DMSO control. In all concentrations of tested rotenone, 10 mg/mL ascorbic acid lowered the ROS but the difference was never statistically significant (Figure 10).

## 4. Discussion

In our cells 150 μM rotenone disrupts the actin and microtubule cytoskeleton, increases ROS but does not significantly alter ATP levels. Additional effects include a stimulation of mitochondrial velocity and an inhibition of mitochondrial fusion. While many studies have similarly shown these results, ours is the first study to explore the effect of rotenone on *D. discoideum* mitochondrial dynamics. Mitochondrial dynamics determine mitochondrial structure, location, and function. In an effort to contribute to understanding the mechanism of Parkinson’s disease we are looking upstream of mitochondrial dysfunction to determine if dynamics play a significant role.

### 4.1. Rotenone Toxicity

Dopaminergic cells are sensitive to rotenone [38] and it is toxic to *D. discoideum* cells with significant death occurring at concentrations greater than 200 μM (data not shown). Rotenone is known to increase ROS, likely thru inhibition of complex I of the electron transport chain [44]. As expected ROS was significantly increased in our cells, also likely due to an inhibition of complex I. Interestingly, the increase of ROS plays a major role in regulating cellular processes [46], including altering ATP levels. Several studies have seen a decrease in ATP levels [44,47]. In our cells we did not see a significant change to ATP levels, though ATP did slightly increase. A study by Annesley et al., 2016 with lymphocytes from Parkinson’s patients also saw an increase in ATP levels [48]. The literature suggests that the change in ATP levels is in itself not toxic to the cells [44]. Additionally, rotenone toxicity can be attributed to loss of the cytoskeleton [37,49]. Beyond the disruption of transport, evidence indicates that loss of microtubules in dopaminergic cells decreases dopamine transport thus the excess dopamine oxidizes forming ROS [50]. In addition to the disruption of microtubules, we and others show that rotenone alters the actin cytoskeleton as well [49]. In conclusion, rotenone toxicity in *D. discoideum* is likely due to the disruption of microtubules and actin filaments altering transport and motility as well as the increase in ROS causing cellular damage leading to death. These traits have been identified in PD cells suggesting that *D. discoideum* could be a cellular model system for PD.

### 4.2. Rotenone and Mitochondrial Dynamics

Our past studies show the importance of the cytoskeleton in terms of mitochondrial dynamics. Specifically the loss of microtubules causes a decrease in mitochondrial velocity, fission, and fusion, while loss of actin decreases the percentage of moving mitochondria [14]. In this work, rotenone affects dynamics but not equivalently to the simple loss of the cytoskeleton. Rotenone instead unbalances fission and fusion, by significantly decreasing fusion, and increasing mitochondrial velocity, with no effect on the percentage of moving mitochondria. These effects are likely due to a combination of cytoskeletal defects and the increase in ROS. Perhaps the direct effect is on fusion; therefore indirectly velocity is increased in an effort for the mitochondria to find each other to maintain the fusion processes. Alternatively, the disruption of the mitochondria’s main transport mechanism could decrease the ability of two organelles to effectively come in contact with each other thus decreasing fusion. Interestingly, Peng et al., 2017 saw a similar phenotype in PC12 cells with expression studies indicating an increase in fission proteins and a decrease in fusion proteins [51].

### 4.3. Rotenone’s Effect on Dynamics Is Not Specific to ROS

Sherer et al., 2003 suggest that prevention of rotenone induced oxidative damage is protective [44]. We tested this in *D. discoideum* by the addition of the antioxidant ascorbic acid to cells previously treated with rotenone. Our results indicate a recovery of the cytoskeleton structure and fusion rates, but an even faster rate of mitochondrial motility. Analysis of ATP levels again indicates no significant difference though levels do trend back toward levels found in untreated cells. Interestingly, ROS levels decreased when exposed to ascorbic acid as compared to rotenone, but not significantly. The antioxidant either did not efficiently remove ROS or was not used at a high enough concentration, though 10 mg/mL is well beyond that typically used in *D. discoideum* studies [45].

Surprisingly ascorbic acid returned the cytoskeleton back to its fully assembled form. It is known that rotenone binds to microtubules preventing assembly and inducing aggregation [34,40,50]. The use of ascorbic acid to recover assembly would seem to suggest that ROS disrupts the cytoskeleton, but it is more likely that ascorbic acid itself induces microtubule assembly as suggested by Boxer et al., 1979 [52]. Rotenone also alters actin structure by stabilizing/blocking dynamics via the Rho GTPases [49], whether this is direct or indirect via ROS is unclear at this time. Ascorbic acid is synergistic with rotenone in terms of mitochondrial velocity. There is precedence for increased cell motility and microtubule trafficking organelles in the presence of ascorbic acid [52], thus we suggest that rotenone induced changes in velocity are due to the disruption of the cytoskeleton not ROS.

The recovery of fusion events suggests that rotenone inhibits fusion via the increase in ROS or via cytoskeleton destruction. Our experiments do not determine whether this inhibition is due to a signaling for fusion to stop, because of a build-up of oxidative damage, or because the tracks for motility are absent. Yet it is intriguing that this study is the first in *D. discoideum* to show an unbalancing of fission, fusion, and motility. Past studies indicate that mitochondrial dynamics are intimately interlinked, if one is inhibited others are as well [11,14]. Thus far it has not been identified which processes drive the others. Through the use of rotenone and ascorbic acid our work suggests that fission and fusion are not as dependent on mitochondrial velocity as previously thought, as they did not speed up when velocity did.

### 4.4. Parkinson’s Disease

Rotenone is implicated as the cause or contributing factor in some cases of PD, especially in rural, agriculture areas. In an effort to understand PD at the cellular level and the role of mitochondrial dynamics in PD, we analyzed mitochondrial dynamics, ATP and ROS levels in amoeba after treatment with rotenone. Our results suggest that *D. discoideum* may be a valid cellular PD model and that the rotenone induced velocity increase along with the loss of fusion could prevent mitochondria from effectively providing energy and other mitochondrial products in high demand areas. The combination of the defects and increased ROS could cause an increase in damaged mitochondria and thus cell death. Neurons are the most susceptible cells to defects in mitochondrial dynamics, thus it is possible for defects like we report here to result in degeneration of neurons in the substantia nigra. New classes of compounds called mitochondria-targeted antioxidants have been developed in the last 18 years and have shown some promise in experimental models by reducing reactive oxygen species [53,54]. Use of ascorbic acid recovered fusion, but exacerbated the motility defect. Further research into the interdependence of mitochondrial dynamics is needed but this study suggests that mitochondrial dynamics may be more important in the progression of PD than previously realized.
